# Automated brain tumour detection and segmentation using superpixel-based extremely randomized trees in FLAIR MRI

**DOI:** 10.1007/s11548-016-1483-3

**Published:** 2016-09-20

**Authors:** Mohammadreza Soltaninejad, Guang Yang, Tryphon Lambrou, Nigel Allinson, Timothy L. Jones, Thomas R. Barrick, Franklyn A. Howe, Xujiong Ye

**Affiliations:** 1 Laboratory of Vision Engineering, School of Computer Science, University of Lincoln, Lincoln, LN6 7TS UK; 2 Neurosciences Research Centre, Molecular and Clinical Sciences Institute, St. George’s, University of London, London, SW17 0RE UK; 3 National Heart and Lung Institute, Imperial College London, London, SW7 2AZ UK; 4 Atkinson Morley Department of Neurosurgery, St George’s Hospital London, London, SW17 0RE UK

**Keywords:** Brain tumour segmentation, Extremely randomized trees, Feature selection, Magnetic resonance imaging, Superpixels, Textons

## Abstract

**Purpose:**

We propose a fully automated method for detection and segmentation of the abnormal tissue associated with brain tumour (tumour core and oedema) from Fluid- Attenuated Inversion Recovery (FLAIR) Magnetic Resonance Imaging (MRI).

**Methods:**

The method is based on superpixel technique and classification of each superpixel. A number of novel image features including intensity-based, Gabor textons, fractal analysis and curvatures are calculated from each superpixel within the entire brain area in FLAIR MRI to ensure a robust classification. Extremely randomized trees (ERT) classifier is compared with support vector machine (SVM) to classify each superpixel into tumour and non-tumour.

**Results:**

The proposed method is evaluated on two datasets: (1) Our own clinical dataset: 19 MRI FLAIR images of patients with gliomas of grade II to IV, and (2) BRATS 2012 dataset: 30 FLAIR images with 10 low-grade and 20 high-grade gliomas. The experimental results demonstrate the high detection and segmentation performance of the proposed method using ERT classifier. For our own cohort, the average detection sensitivity, balanced error rate and the Dice overlap measure for the segmented tumour against the ground truth are 89.48 %, 6 % and 0.91, respectively, while, for the BRATS dataset, the corresponding evaluation results are 88.09 %, 6 % and 0.88, respectively.

**Conclusions:**

This provides a close match to expert delineation across all grades of glioma, leading to a faster and more reproducible method of brain tumour detection and delineation to aid patient management.

## Introduction

Despite improvements in the diagnosis and oncological treatment of primary brain tumours, they remain associated with significant morbidity and a poor overall prognosis. The majority of primary brain tumours originate from glial cells (termed glioma) and are classified by their histopathological appearances using the World Health Organization (WHO) system into low-grade glioma (LGG) (grades I and II) and high-grade glioma (grade III anaplastic glioma and grade IV glioblastoma). The typical natural history of low-grade glioma is a latent period of growth and infiltration of white matter with subtle neuro-cognitive deficit and seizures in some cases followed by regional change or transformation to a more malignant variant. High-grade glioma may present as a de novo (primary) glioblastoma or as a transformation of a lower-grade tumour (e.g. secondary glioblastoma).

Gliomas typically originate within white matter and exhibit irregular growth patterns along white matter fibres, infiltrating surrounding brain. As a result, they exhibit irregular boundaries that may be visually indistinct on conventional magnetic resonance images. Delineation of the tumour boundary and assessment of tumour size are needed for patient management in terms of treatment planning and monitoring treatment response, and current guidelines incorporate the use of both contrast-enhanced T1-weighted (CE T1w) images and T2-weighted (T2w) / FLAIR images [1, 2]. Many low- grade gliomas do not show contrast enhancement; hence, T2w/FLAIR images are used to define the tumour extent and volume. A longitudinal study has shown that LGG volume and growth rate can be used to assess whether patients are at risk with tumours likely to undergo an early malignant transformation [3]. In clinical studies, current Response Assessment in Neurooncology (RANO) criterion simply uses a bidirectional measurement to determine tumour volume for assessing treatment response [4]. Although a full 3D volume measurement may provide a more accurate volume assessment, there is a need for accurate and fully automated methods since manual segmentation (region of interest drawing) around tumour margins on a slice-by-slice basis is time-consuming and can take 12 min or more per tumour, with semiautomatic methods taking 3–5 min [5, 6]. T2w/FLAIR images can also be useful to help define the target volumes for radiotherapy planning of high-grade gliomas [2, 5]; hence, an automated segmentation that is not subject to operator subjectivity may be beneficial [5]. In this study, we have concentrated on developing and validating an automated method for a single MRI modality, FLAIR, that could be readily translated for clinical use. Future automated methods are likely to incorporate information from multimodal clinical MRI as in the Multimodal Brain Tumor Image Segmentation Benchmark (BRATS) database studies [7–9] and also include perfusion and diffusion imaging to detect tumour tissue subtypes (e.g. necrosis, active tumour, infiltrative tumour, oedema) [10].

However, automated detection and segmentation of brain tumour is a very challenging task due to its high variation in size, shape and appearance (e.g. image uniformity and texture) [11]. Also, typical clinical image acquisition protocols usually lead to higher intraslice resolution than interslice resolution to achieve the balance of good apparent image resolution with adequate signal to noise and restricted scanning time that causes asymmetry in partial-volume effects. High-grade gliomas usually have irregular boundaries which, in some cases, are unclear or discontinuous [12]. Current work on brain tumour segmentation can be categorized into atlas-based [13–15], unsupervised [16–19], hybrid [20–22] and supervised- based approaches [23–26].

In Ref. [20], a hybrid method was proposed for brain tissue detection in MRI images which included seeded region growing segmentation and neural network classification. However, the method was semiautomatic and different parts of tumour need to be pre-selected to initiate the segmentation process. Another method is proposed for detection of multiple sclerosis (MS) lesions in brain MR images which consisted of rule-based, level-set and support vector machines [21]. Rajendran and Dhanasekaran [22] proposed a hybrid method for segmenting the tumour by combining region-based fuzzy clustering and deformable model. However, the method was only applied on a few FLAIR images with fixed parameters.

Supervised learning-based algorithms use training data labelled by experts for segmentation of tumours. Geremia et al. [23] used discriminative random decision forests to classify the voxels of 3D MRI image for segmentation of MS. Wu et al. [24] used superpixel features in a conditional random fields (CRF) framework to detect brain tumours. However, the method was not satisfactory for low-grade tumours segmentation. A method was proposed in [25] which used extremely randomized forest classification considering both appearance and context-based features. Another method was proposed in [26] which used ERT for classification of voxels based on their statistical and textural features, which were extracted using different MRI protocols. In order to reduce the computation time, it was suggested that features were only extracted from a random set of voxels, but this resulted in losing some part of data. In addition, a fixed size neighbourhood for each voxel was used to calculate features.

A number of advanced algorithms [23, 27–31] were recently presented in [7] using the BRATS [8, 9] organized in conjunction with the international conference on Medical Image Computing and Computer-Assisted Interventions (MICCAI) 2012 and 2013 conference. The methods were based on segmentation of different tumour tissues, i.e. tumour core, oedema, necrosis, using multiprotocol containing FLAIR, T1-weighted (T1w), T1w with contrast and T2-weighted protocols [32].

Despite much effort being devoted to the segmentation problem, brain tumour segmentation remains an ongoing research topic. Very few completely automatic segmentation algorithms have been adopted in the clinic. Recently, only one automated tool has been clinically evaluated [33].

In this study, we investigate a fully automated superpixel-based method for detection and segmentation of the abnormal tissue associated with brain tumours as defined by the $\hbox {T}_{2}$ hyperintensity from Fluid-Attenuated Inversion Recovery (FLAIR) MRI. FLAIR images are routinely acquired as part of standard diagnostic clinical MRI of brain tumours. Delineation of the FLAIR hyperintensity is relevant for assessing low-grade glioma growth [34], defining an abnormality region from which imaging features for tumour classification can be extracted [35], aiding with radiation dose planning [36] and assessing treatment response [37]. Different from the methods in [25] and [26], in which image features were calculated based on each individual voxel and a fixed size neighbour-hood was considered for the feature extraction, in this paper, superpixel partition is firstly calculated which provides accurate boundaries between different tissues, and then image features are extracted from each superpixel. This will not only improve the accuracy of feature calculation, but also increase the speed of computation. We demonstrate the automated method that provides a close match to expert delineation across all grades of glioma and so could provide a faster and more reproducible method of brain tumour delineation to aid patient management. To assess the robustness of the proposed method, the method is also evaluated on the FLAIR protocol of BRATS 2012 annotated training dataset [8, 9].

The rest of this paper is organized as follows. The “Method” section describes the proposed method, including superpixel partition, feature extraction, classification and final segmentation. The “Experimental results” section presents the data description and experimental results for the two datasets, followed by “Discussion” and “Conclusion” sections.

## Method

**Fig. 1:**
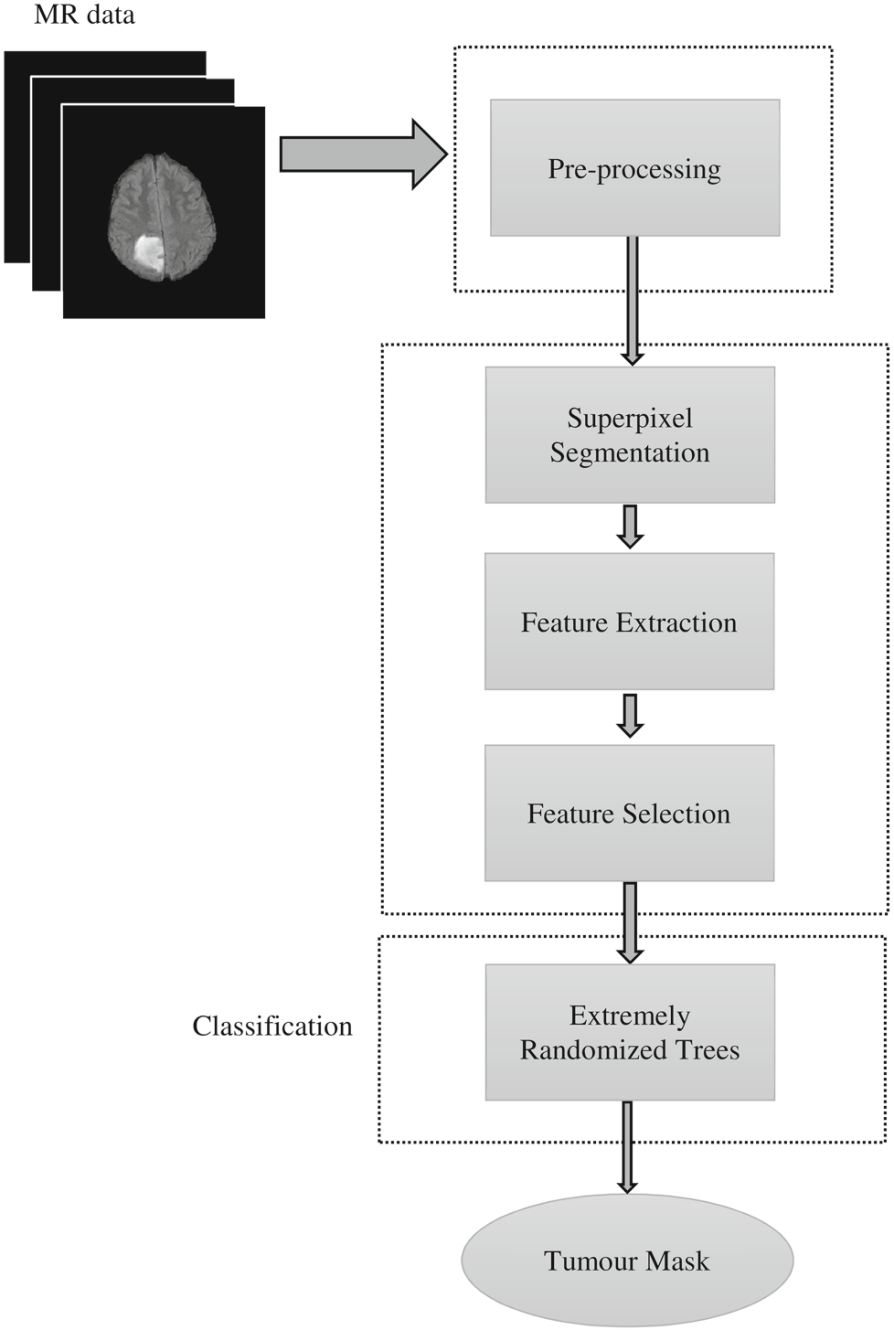
Flowchart of the proposed method

Our method consists of four main steps, which are depicted in Fig. 1. After preprocessing, in the superpixel segmentation step, FLAIR image is partitioned into irregular patches with approximately similar size and intensity values. For each superpixel patch, a number of features including statistical, texton and shape features are calculated. This is then followed by feature selection to find the most significant features, based on which each superpixel is classified into tumour and non-tumour using an ERT classifier.

### Preprocessing

First, the skull is removed from all the MRI images using FSL [38]. Then, histogram matching algorithm [39] is applied to ensure that all the data have similar dynamic ranges. ITK software [40] is used for this task, and one of the cases is selected as the reference, and then, other MRI FLAIR scan intensities are transformed to match the histogram of the reference image.

**Fig. 2:**
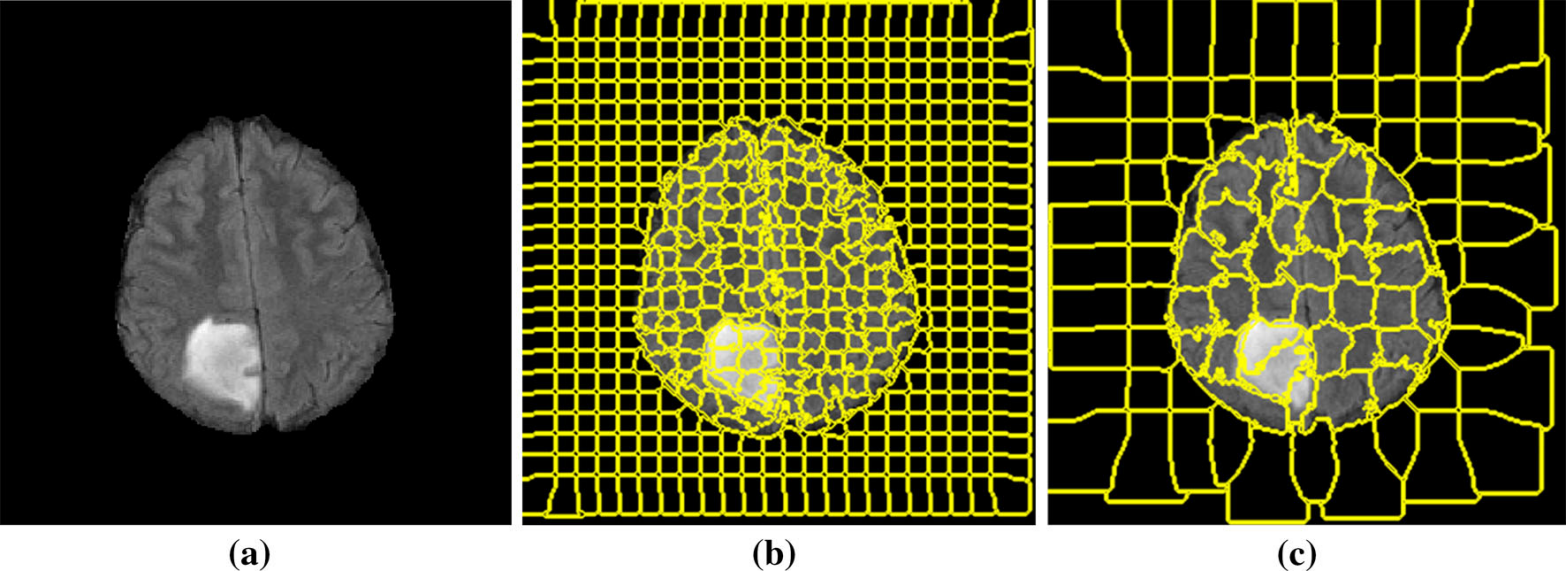
Example of superpixel segmentation with different window sizes: **a** original MRI FLAIR image with a grade II tumour, **b** superpixel segmentation with $S= 10$ (initial grids $10\times 10$) and $m = 0.2$, **c** superpixel segmentation with $S = 20$ (initial grids $20\times 20$) and $m = 0.2$

### Superpixel segmentation

The simple linear iterative clustering (SLIC) [41] method is used to partition the image into patches with approximately similar size. SLIC method has a few parameters which are flexible to be tuned by controlling the trade-off between them and boundary adherence. Furthermore, it is computational and memory efficient. Each slice of FLAIR image is gridded into equally sized squares with a user-defined size. The size of grid side for these initial superpixels is considered as *S*. The geometrical centre of each segment is considered as the superpixel centre. These centre coordinates are then updated in each iteration. The pixels are grouped based on their spatial and intensity distance metrics. The spatial distance $d_\mathrm{s}$ between the $i\hbox {th}$ pixel and the $j\hbox {th}$ pixel is calculated as:

1 $$\begin{aligned} d_\mathrm{s} =\sqrt{\left( {x_j -x_i } \right) ^{2}+\left( {y_j -y_i } \right) ^{2}} \end{aligned}$$

where *x* and *y* are the pixel location coordinates. The intensity distance $d_\mathrm{c}$ between the two pixels is defined as:

2 $$\begin{aligned} d_\mathrm{c} =\sqrt{\left( {I_j -I_i } \right) ^{2}} \end{aligned}$$

where $$I_{i}$$ and $$ I_{j}$$ are the normalized intensity values of the $$i\hbox {th}$$ and the $$j\hbox {th}$$ pixel, respectively.

The overall distance measure which is a combination of spatial and intensity distances is then calculated with:

3 $$\begin{aligned} D=\sqrt{d_\mathrm{c}^2 +\left( {\frac{d_\mathrm{s} }{S}} \right) ^{2}m^{2}} \end{aligned}$$

where *m* is a compactness coefficient which determines the flexibility of superpixel boundaries. A higher value of *m* results in more compact segments and a lower value creates more flexible boundaries. It is noted that, to obtain an optimum compactness coefficient *m*, the MRI image intensities used in Eq. (2) are normalized to the values of [0, 1]. This is to ensure that both the intensity and space distances are within the same range.

Figure 2 shows MR images acquired with protocol FLAIR containing a grade II tumour which is partitioned to superpixels with two different side sizes, *S*. The compactness factor *m* is set to be 0.2 for both sizes. In Fig. 2b, c, the superpixels are extracted with $S = 10$ and $S = 20$, respectively.

### Feature extraction and selection

In order to train a robust classifier for the detection and segmentation of brain tumour, different types of features are considered, including intensity statistics, textons and curvature features.

#### Intensity statistical features

First-order intensity statistics [42] are referred as pixel intensity-based features. They express the distribution of grey levels within the selected region of interest (ROIs) which are the superpixels in our work. For each superpixel, 16 features are calculated which are average, standard deviation, variance, mean of the absolute deviation, median absolute deviation, coefficient of variance, skewness, kurtosis, maximum, minimum, median and mode of the intensity values, central moments, range, interquartile range and entropy.

**Fig. 3:**
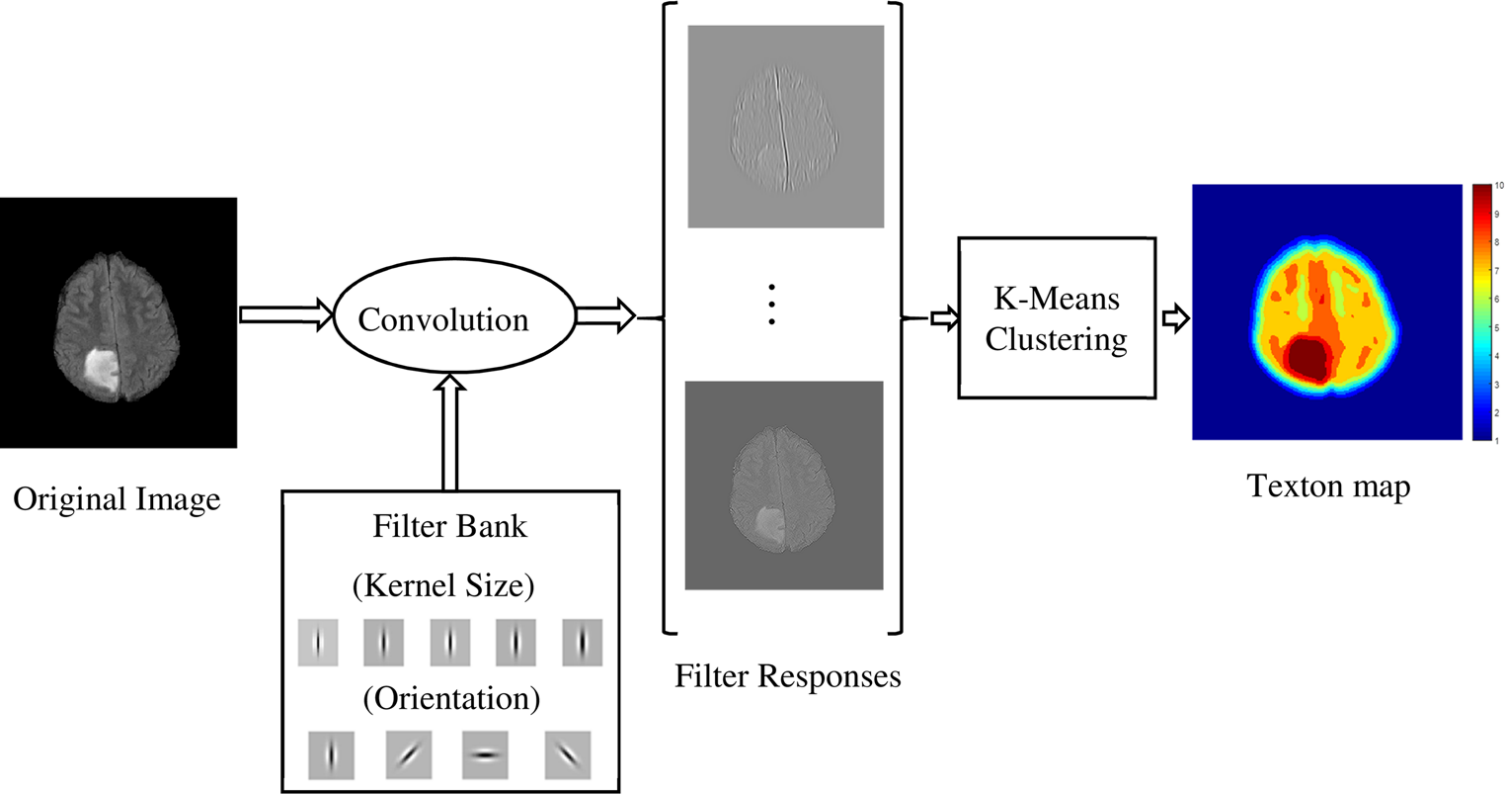
Procedure of texton feature extraction using Gabor filters applied to a grade II glioma

#### Texton feature

Brain tissues have complex structures, so the intensity features are not sufficient for accurate segmentation of tumour. Texture features are used to improve the accuracy of segmentation. In this study, the texture features are calculated based on texton analysis. Textons are small elements of the image generated by convolution of the image with a specific filter bank, in which Gabor filter [43] defined as Eq. (4) is used:

4 $$\begin{aligned}&G\left( x,y;\theta ,\sigma ,\lambda ,\psi ,\gamma \right) \nonumber \\&\quad =\hbox {exp}\left( -\frac{x^{{\prime }2}+\gamma ^{2}y^{{\prime }2}}{2\sigma ^{2}} \right) \hbox {exp}\left( i{\left( 2\pi \frac{x^{\prime }}{\lambda }+\psi \right) } \right) \end{aligned}$$

where $\sigma$ is the filter size, $\lambda$ is the wavelength of sinusoid, $\psi$ is the phase shift and $\gamma$ is the spatial aspect ratio. In Eq. (4), the terms ${x}^{\prime }$ and ${y}^{\prime }$ are calculated from the spatial orientation of the filter, $\theta$, defined as:

5 $$\begin{aligned} x^{\prime }= & {} x\cos \theta + y\sin \theta \nonumber \\ y^{\prime }= & {} x\cos \theta + y\sin \theta \end{aligned}$$

The values which are set for these parameters will be discussed in “Texton feature parameters” section.

The FLAIR image is convolved with all the $N_\mathrm{FB}$ filters (i.e. $N_{\mathrm{FB}}$ is the number of filters in the filter bank) and a response vector with length of $N_{\mathrm{FB}}$ is generated for each pixel. These filter response vectors (the number of vectors is the same as the number of the pixels in the image) are then clustered into *k* clusters using $N_{\mathrm{FB}}$-dimensional k-means clustering. The filter response vectors corresponding to each cluster are considered as the texton of a particular texture class. By assigning the cluster number to each pixel, a texton map of the image is obtained. The procedure of texton map extraction is depicted in Fig. 3. The texton features for each superpixel are then calculated using the histogram of texton map within each superpixel.

#### Fractal features

A segmentation-based fractal texture analysis method (SFTA) [44] is used to calculate fractal features. The image is firstly decomposed into a set of binary images based on multilevel thresholds computed using Otsu algorithm [45]. The desired number of thresholds $n_\mathrm{t}$ is defined by the user (in this paper, $n_\mathrm{t} = 3$). Then for each binary channel, all the image boundaries are extracted using edge detection [46]. The fractal features are calculated from these binary edge channels which include area, intensity and fractal dimension. Area feature is the number of edge pixels in a superpixel. Intensity feature is the mean intensity of image pixels corresponding to the edge pixels in a superpixel. Fractal dimension represents the complexity of the structure of the image and is calculated from image boundary as:

6 $$\begin{aligned} D_0 =\mathop {\lim }\limits _{\varepsilon \rightarrow 0} \frac{\log N\left( \varepsilon \right) }{\log {\varepsilon }^{-1}} \end{aligned}$$

where $N(\varepsilon )$ denotes the counting of hypercubes of dimension *E* and length $\varepsilon$. By using box counting algorithm [47], an approximation of fractal distance is obtained from the binary images.

**Fig. 4:**
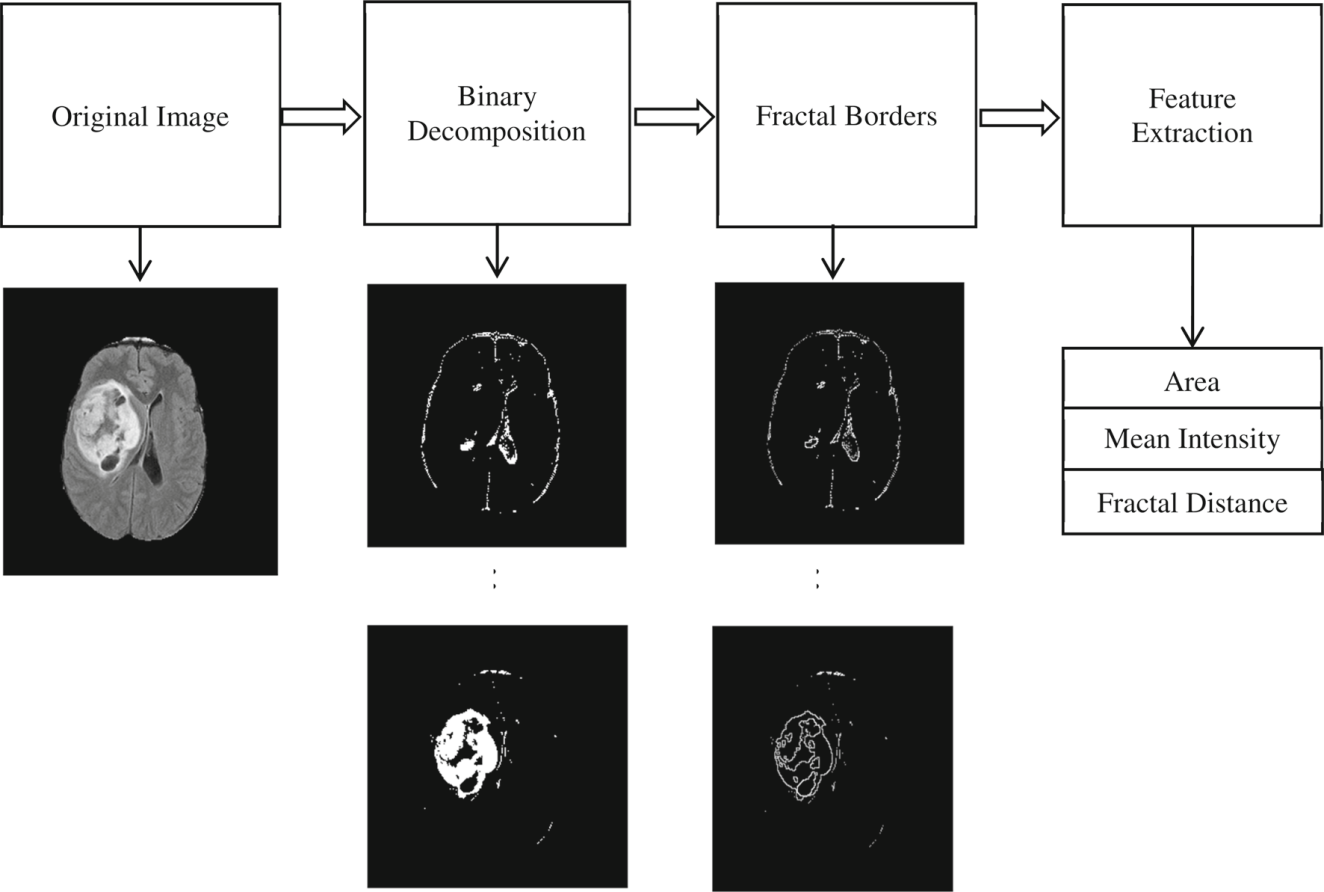
The flowchart of extracting fractal features from a grade III glioma

**Fig. 5:**
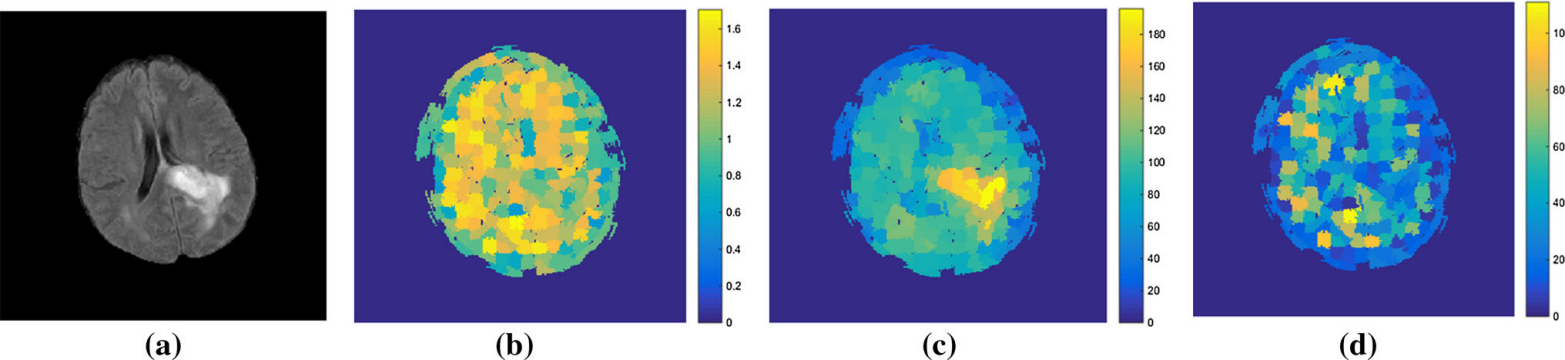
An example of fractal analysis applied to a grade III glioma to generate superpixel-based fractal feature maps: **a** FLAIR image, **b** area, **c** mean intensity and **d** fractal dimension

**Fig. 6:**
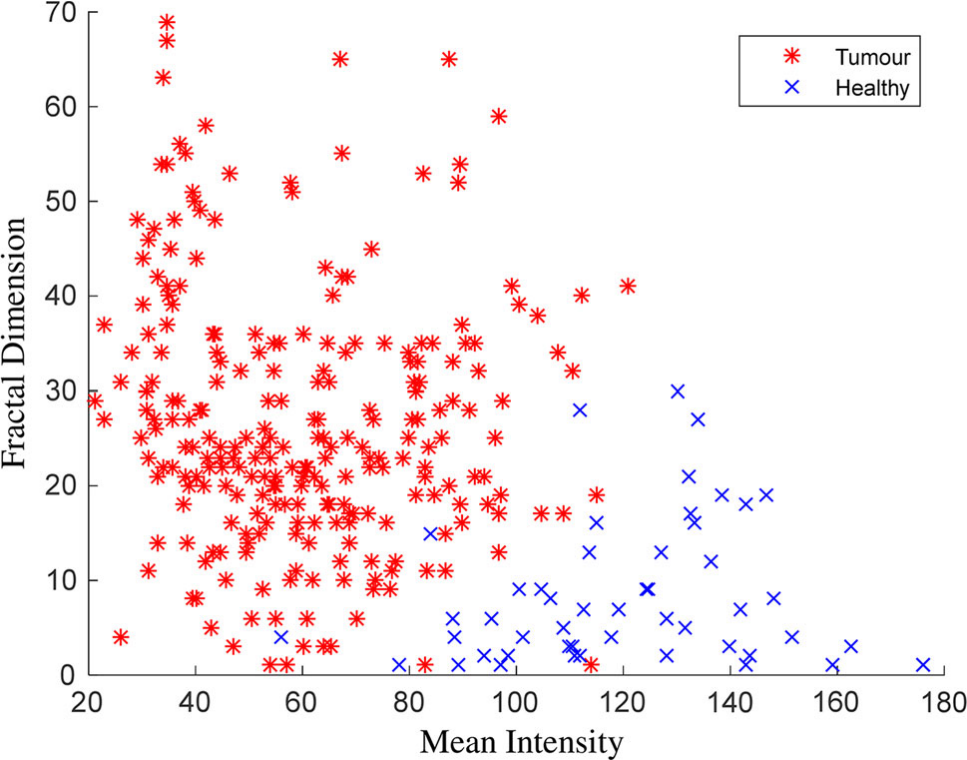
Fractal dimension versus mean intensity for healthy and tumour superpixels calculated from one FLAIR MRI data with grade IV glioma

Figure 4 presents a flowchart of fractal analysis. Figure 5 shows fractal features including: area, mean intensity and fractal dimension. Figure 6 illustrates an example of fractal dimension and mean intensity features calculated from healthy and tumour superpixels from one patient data containing a grade IV glioma. It demonstrates a good separation in feature space (mean intensity fractal dimension) for FLAIR images.

#### Curvature feature

Image curvature is a shape-based feature which is computed by the gradients along *x* and *y* directions of an image, namely $f_{x}$ and $f_{y}$. The image normal at pixel ($x,\, y)$ is then calculated as [48]:

7 $$\begin{aligned} \hat{{{\varvec{N}}}}\left( {x,y} \right) =\frac{1}{\left( {f_x^2 +f_y^2 } \right) ^{1/2}}\left( {{\begin{array}{l} {f_x } \\ {f_y } \\ \end{array} }} \right) . \end{aligned}$$

The two-dimensional curvature of the image is the divergence of this normal and is calculated as:

8 $$\begin{aligned} \hbox {Curv}=\frac{f_{xx} f_y^2 +f_{yy} f_x^2 -2f_{xx} f_x f_y }{\left( {f_x^2 +f_y^2 } \right) ^{3/2}} \end{aligned}$$

where $f_{xx}$ and $f_{yy}$ are the second derivatives of the image intensity *I*(*x*, *y*). The curvature feature for each superpixel is the average of the curvature values for all the pixels in the superpixel.

In summary, there are in total 28 features calculated for each superpixel, among which there are 5 texton histogram features from 5 clusters and 6 fractal features obtained from 3 thresholded binary images (each binary image provides 3 fractal features). It is noted that all the features, except the 5 texton histogram features, are normalized to the range of [0,30], and this is to ensure that all the features have similar dynamic ranges and also are close to the textons histogram values. Table 1 shows a list of the features. The details of parameter setting in feature calculation will be discussed in “Selection of parameters” section.

#### Feature selection

Feature selection step is used not only to increase the computation speed, but also to remove redundant features which may cause more classification error. In this paper, we employ the Minimum Redundancy Maximum Relevance (mRMR) feature selection technique proposed by [49]. mRMR is an efficient technique for subset selection of features, which selects more relevant features by removing the irrelevant ones. Mutual information is used for identifying the similarity between features. For features, $f_{i}$, in feature set *S*, the maximum relevance is obtained between features and class *c* by maximizing the following:

9 $$\begin{aligned} \max D\left( {S,c} \right) , \quad D=\frac{1}{\left| S \right| }\mathop {\sum }\nolimits _{f_i \in S} I_\mathrm{M} \left( {f_i ;c} \right) \end{aligned}$$

where $I_\mathrm{M}$ is mutual information between feature $f_{i }$ and the class *c*. Minimum redundancy is calculated from:

10 $$\begin{aligned} \min R\left( s \right) ,\quad R=\frac{1}{\left| S \right| ^{2}}\mathop {\sum } \nolimits _{f_i ,f_i \in S} I_\mathrm{M} \left( {f_i ,f_j } \right) . \end{aligned}$$

The feature selection is performed on the entire feature vector, and it is based on leave-one-out cross-validation using voting scheme. For each case, using cross-validation, the best $N_\mathrm{FEA}$ features were selected. It is noted that, if one feature is selected by one case, the feature will get one vote. For all the features voted by all the cases, the top $N_\mathrm{FEA}$ features with highest scores will be chosen as the final features. The selected features will be used in the classification stage to classify each superpixel into tumour or non-tumour.

**Table 1:** Total number of features calculated from MRI FLAIR image

Feature name	Number of features
Statistical 1st order	16
Texton histogram	5
Fractal	6
Curvature	1
Total	28

### Extremely randomized tree-based classification of superpixels

In order to tackle the problem of extremely imbalanced data in our dataset, ERT classifier [50] is used to categorize each superpixel into tumour or normal brain tissue and to improve the accuracy of the minority class (e.g. tumour). Like random forests (RF) [51], ERT is an ensemble technique which uses multiple decision trees. For both methods, each node of the tree includes a set of training examples and the predictor. Splitting starts from the root node and will continue at every node. The procedure is performed based on the feature representation and allocating the partitions to the right and left nodes. Tree grows until a specified tree depth. During the bagging process and at each attribute split, a random subset of features is used. In RF, by generating large number of trees, the most popular class is voted [52].

ERT is an extension of RF in which a further randomization stage is added for selecting the cut-points alongside with randomized selection of attributes like in RF. In this technique, the splits of attributes and cut-points are selected randomly. Each tree is determined by $t \epsilon \{1{\ldots } T\}$ in which *T* is the number of randomized trees. For a given data point *x* and dataset $D_\mathrm{train}$, a feature vector is represented by $f(x, D_\mathrm{train})$. To classify the class *c* of the data, for an n-dimensional feature representation, each tree learns a weak predictor of $p_{t} (c{\vert }f(x, D_\mathrm{train}))$.

In the testing process, for an unseen data point, $x'$, the probability of belonging to a class *c* is calculated by the average of probabilities on all the trees:

11 $$\begin{aligned} p\left( {c\hbox {|}f\left( {x^{\prime },D} \right) } \right) =\frac{1}{T}\mathop \sum \limits _{t=1}^T p_t \left( {c\hbox {|}f\left( {x^{\prime },D} \right) } \right) \end{aligned}$$

The structures of randomized trees are independent of training sample outputs. The parameters should be selected and tuned for the specific case. In our method, there are 20 extra trees in the ensemble and five attributes, which are equal to the number of selected features, are selected to perform the random splits. Tree depth is chosen to be 15 and the minimum number of samples for splitting a node is 2 as this is a classification task. Setting these parameters will be discussed in “Extremely randomized trees parameters” section.

After the ERT, each superpixel is then classified into tumour or non-tumour candidates. For all the tumour superpixels, a 3D connected component analysis [53] is then used to obtain 3D connected superpixel regions. Each small superpixel region in which the total number of voxels in the region is less than a pre-defined threshold (i.e. 100) is regarded as a false-positive (FP) region and removed from the tumour candidates. The remaining tumour superpixel regions are the segmented tumour.

**Fig. 7:**
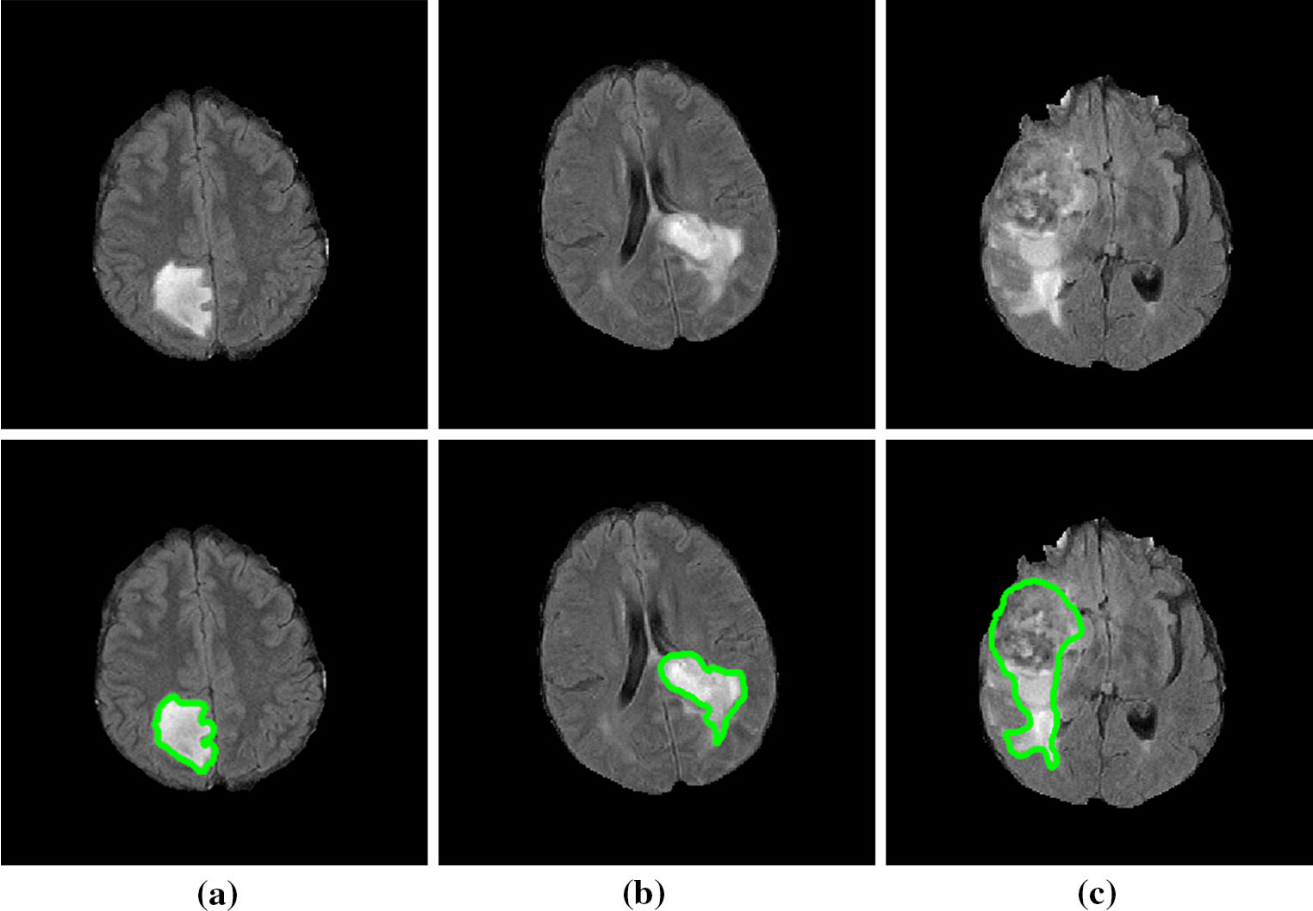
FLAIR images with different tumour grades in *upper row* and their ground-truth manual segmentation of the FLAIR hyperintensity in the *lower row*. Tumour grades are: **a** grade II, **b** grade III and **c** grade IV

## Experimental results

Two experiments were carried out in this section. In the first experiment, our own clinical dataset is used for training and validation of the algorithm. In the second experiment, the method is further validated on the publicly available MICCAI BRATS 2012 dataset [7–9] to assess the robustness of the method. The following subsections, including data description, parameters selection and comparative experimental results are focused on our own data cohort, while the next subsection presents the evaluation results on MICCAI BRATS 2012 clinical training dataset.

### Data description

We acquired patient data using a GE Signa Horizon LX 1.5 T MRI system (GE Healthcare, Milwaukee, WI, USA) equipped with a maximum field gradient strength of 22 mT/m and using a quadrature head coil. The MRI sequence used in this study is FLAIR which is acquired in the axial plane with a field of view (FOV) $240\times 240\,{\hbox {mm}}^2$, matrix size $256\times 256$ and 5 mm slice thickness with no slice gap. In particular, the following sequence is used: FLAIR (TE$\,=\,133$ ms, TR$\, =9000$ ms, inversion time 2200 ms).

A cohort consisting of 19 patients entered retrospectively into our study, each with a brain tumour, who has been imaged with the FLAIR MRI sequences. The dataset consists of 6 grade II tumours, 3 grade III tumours and 10 grade IV tumours. Each patient has a histological gold standard of tumour grading. Figure 7 shows some examples of the manual segmentations for different tumour grades in FLAIR images. Patient ages at the time of scanning ranged from 22 to 73 (mean 54) and consisted of 7 females and 12 males.

### Selection of parameters

Statistical features are calculated directly from the intensity values of the pixels within the superpixels, and they are nonparametric. Parameter setting is required to calculate texton and fractal features. For texton features, parameters of Gabor filter bank and the number of clusters in k-means clustering need to be determined. For the ERT classifier, an optimum number of trees should be selected for an accurate and fast classification. In this study, the parameters are determined through the training stage, in which a total number of 6 patients’ data are randomly selected including 2 grade II, 1 grade III and 3 grade IV. In the following section, the process of these parameters selection is explained in detail.

#### Superpixel parameters

**Fig. 8:**
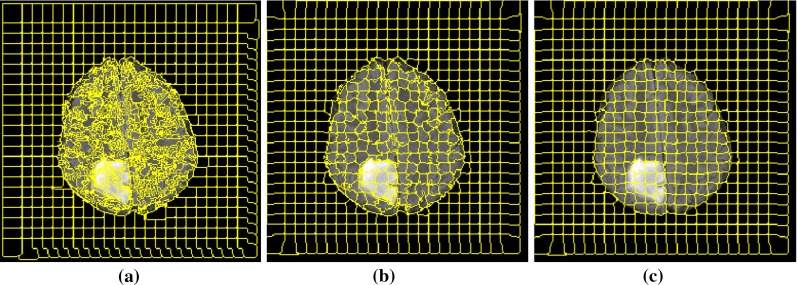
Superpixel segmentation with $S= 10$ and different compactness: **a** $m = 0$, **b** $m= 0.2$ and **c** $m = 0.5$

To investigate the effect of compactness factor, *m*, defined in Eq. (3), on the superpixels boundaries, we apply different values from 0 to 1 and inspect the results visually. The intensity values of the FLAIR voxels within the brain are normalized to the range of [0, 1]. A compactness factor $m = 1$ results in more rigid boundaries, while $m = 0$ produces very flexible boundaries but increases the variation and irregularity of the superpixels shapes. An example of this parameter is shown in Fig. 8. By visually inspecting the superpixel boundaries and area, the value of $m = 0.2$ is chosen, which presents coherent boundaries.

To select an appropriate superpixel size, different initial window side sizes are considered in the partitioning stage. The compactness factor is fixed to $m = 0.2$ for all the experiments. Then, the superpixels which have more than 0.9 overlap with the manual segmentation mask are selected and the Dice measure is used for assessing the performance of superpixel segmentation. The experiment ran on the selected training images from different tumour grades. The results are presented in

**Table 2:** Examples of the impact of different initial superpixel side sizes, *S*, on the segmentation accuracy of the tumour in FLAIR images with compactness factor $m = 0.2$

Superpixel side size	4	6	8	10	15	20
Dice overlap	0.98	0.96	0.92	0.85	0.73	0.56

Table 2, which shows that increasing the superpixel size results in less segmentation accuracy. A superpixel size of $S = 6$ is chosen which has a good performance and also contains sufficient information within the superpixel for texture feature calculation.

#### Texton feature parameters

For the direction of Gabor filters, six settings from the range: $[0{^{\circ }},\, 30{^{\circ }}, 45{^{\circ }},\, 60{^{\circ }},\, 90{^{\circ }},\, 120{^{\circ }}]$ are chosen. These degrees cover the whole space of the region with a reasonable step. Although adding more orientations seems to include more details to the features, it will also increase the computation time and may add redundant information which may affect the classification accuracy.

The maximum and minimum values for size and wavelength coefficients are selected empirically in conjunction with visual inspection. For the size values under the 0.3, filtered images are very close to the original image, while for the values above the 1.5, the images are intensively blurred. Therefore, the kernel sizes are selected within this range with the increment of 0.3, i.e. [0.3, 0.6, 0.9, 1.2, 1.5]. Wavelength coefficients are selected empirically by visual inspection of the filters in the range of [0.8, 1.0, 1.2, 1.5].

As discussed in “Texton feature” section, the texton map is created by applying *k*-means clustering to different filter responses. A key question in using *k*-means clustering is to determine the cluster (texton) number *k*. However, it is not straightforward to provide an accurate number of the structures presented in the image. Theoretically, for the texton generation, with the increasing number of clusters, more specific texton differences between clusters could be extracted. However, a large k may result in overclassification and also in increasing computational cost. In our experiment, the number of clusters (textons) ($k=5$) is chosen empirically according to the number of tissues that may be present in the FLAIR images, i.e. grey matter, white matter, tumour, oedema and other tissue types.

#### Fractal feature parameters

**Fig. 9:**
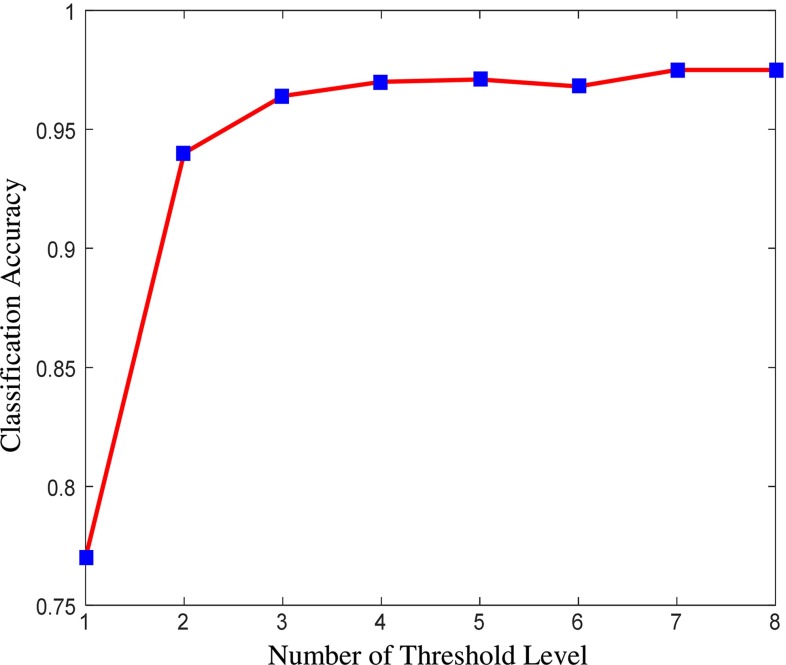
Effect of number of threshold levels on the classification accuracy

Different threshold levels for fractal feature extraction have been examined. The accuracy of superpixel classification using fractal features only is a measure to assess the effect of number of threshold level. As shown in Fig. 9, after increasing $n_\mathrm{t} = 3$ levels of threshold, which creates 6 binary channels, the overlap measure does not increase significantly. On the other hand, increasing each level will add 6 more features (each binary channel has 3 fractal features) to the feature vector. This makes the classification more complicated and also increases the computation time for both fractal feature calculation and classification. Therefore, the optimum level of threshold $n_\mathrm{t} = 3$ is chosen for the segmentation of oedema and tumour core.

#### Extremely randomized trees parameters

Implementation of ERT was performed in MATLAB 2015b using the open- source code provided in [54] which is based on the method by Geurts et al. [50]. To assess the impact of ERT parameters on the classification performance, the experiment ran on the selected training images with different sizes of trees. The maximum depth of the trees for the ERT is set to 15. Minimum sample size, $n_\mathrm{min}$, for splitting a node is selected to be 2 as according to [50] $n_\mathrm{min} = 2$ is an optimal value for most classification tasks. The number of attributes for random split is considered as 5 which is equal to the number of selected features after applying the mRMR feature reduction. As shown in Table 3, by adding more than 20 trees to the ERT, there is no significant improvement for the classifier accuracy. In addition, increasing the number of trees will increase the computation time. Therefore, in our experiment, the size of 20 trees is used for the ERT classifier.

**Table 3:** Impact of the number of trees on ERT classifier accuracy

Number of trees	5	10	20	50	100
Classification accuracy (%)	92.35	97.86	98.22	98.28	98.28

**Table 4:** Comparison evaluation on superpixel classification using SVM-based and ERT-based classifier, respectively, on the 5 features selected using mRMR

Case no.	Grade	SVM			ERT		
		Precision (%)	Sensitivity (%)	BER	Precision (%)	Sensitivity (%)	BER
1	II	62.71	97.33	0.02	69.85	97.45	0.02
2	II	58.65	98.14	0.02	90.24	98.65	0.01
3	II	72.55	98.41	0.02	74.21	99.12	0.01
4	II	68.53	94.88	0.03	70.24	96.05	0.02
5	II	76.33	55.64	0.23	78.43	56.32	0.22
6	II	75.83	73.45	0.14	85.63	71.32	0.15
7	III	84.75	98.75	0.01	86.07	99.35	0.01
8	III	88.54	83.32	0.09	90.78	85.64	0.08
9	III	88.92	98.11	0.01	91.44	98.67	0.01
10	IV	95.22	83.25	0.09	97.44	89.03	0.06
11	IV	93.45	88.53	0.07	96.57	91.65	0.05
12	IV	81.55	73.98	0.14	84.33	75.92	0.13
13	IV	80.35	92.68	0.04	82.53	95.73	0.03
14	IV	90.12	92.51	0.04	91.32	95.87	0.03
15	IV	93.42	93.76	0.04	96.78	94.02	0.03
16	IV	87.45	83.06	0.09	90.21	84.15	0.08
17	IV	95.34	87.75	0.06	96.81	91.87	0.04
18	IV	98.33	82.56	0.09	98.43	85.33	0.08
19	IV	96.21	92.51	0.05	98.12	94.03	0.04
Mean	All	83.59	87.82	0.07	87.86	89.48	0.06
STD	All	11.76	11.09	0.06	9.27	11.23	0.06

*BER* balanced error rate

The classification is performed for tumour including oedema and active tumour core versus normal brain tissue

### Comparative experimental results

Our automated method is compared with the manual annotation provided by an expert. Dice similarity score [55], which calculates the overlap of segmented area and manual segmentation, is used to quantitatively evaluate the proposed method. The Dice overlap measure ranges from 0 to 1. The lower value represents lower overlap, while 1 demonstrates a complete overlap.

In the classification stage, leave-one-out validation is performed on single-channel MR FLAIR data. The brain MR images are partitioned into superpixels based on Eq. (3) using the initial window side size of $$ S = 6$$ pixels and the compactness factor $$m = 0.2$$. All the superpixels inside the brain area are used for classification. Based on the manual annotation, superpixels are split into two classes: normal tissue and brain tumour including tumour core and oedema. Superpixel with at least 50 % of tumour pixels in manual annotation is considered as a tumour superpixel. The remaining superpixels are labelled as normal. The model is trained based on these two labels. During the testing stage, the trained model is then applied and labels are assigned to all the superpixels inside the brain. The ERT classifier is compared with support vector machine (SVM) [56] for the classification of superpixels. The tumour area is obtained by grouping the superpixels related to tumour class.

In total, five features are used after mRMR feature selection, which are the normalized mean intensity, fractal dimension, two texton channels and mean curvature within the superpixel. It is noted that, though ERT can be directly used as feature selection and classification, to ensure a fair comparison between the ERT and SVM classifiers, the same feature set is considered in this study.

Evaluations have been carried out qualitatively by visual inspection and quantitatively using three classification measures for the detection and the Dice overlap measure for the segmentation. It is noted that, for the standard four classification measures (accuracy, precision, sensitivity, specificity), both accuracy and specificity will give very high values due to the highly imbalanced nature of our data. Therefore, to properly evaluate the classification performance, only precision and sensitivity are considered.

Table 4 presents the evaluation measures for SVM and ERT, respectively. It can be seen that, ERT produces a better classification performance, compared to that of SVM, with an overall classification precision of 87.86 %, sensitivity of 89.48 % and BER of 6 % for ERT, and of 83.59 %, 87.82 % and 7 % for SVM, respectively.

**Fig. 10:**
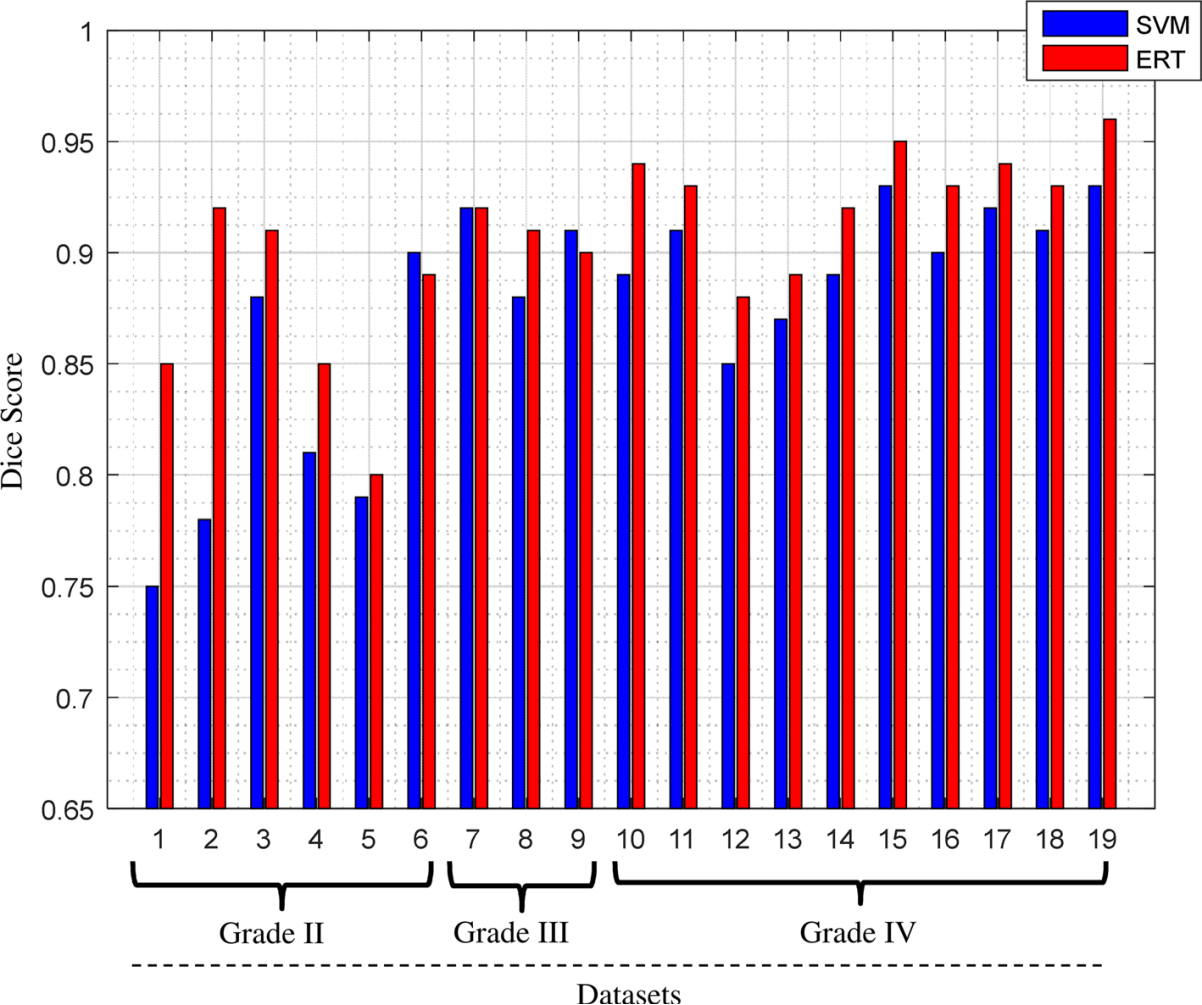
Comparison of Dice score overlap measure of SVM versus ERT for all our clinical patient data (19 scans). Dice score in vertical axis starts from 0.65 for better illustration

The Dice score overlap measure of the individual patient comparing the ground truth with the segmented tumour masks using both SVM and ERT is plotted in Fig. 10. It can be seen that the overlap ratio using the ERT-based method is much better than that of SVM-based for all the three tumour grades, with mean and standard deviation Dice score of 0.91 ± 0.04 for ERT-based and 0.87 ± 0.05 for SVM-based.

**Fig. 11:**
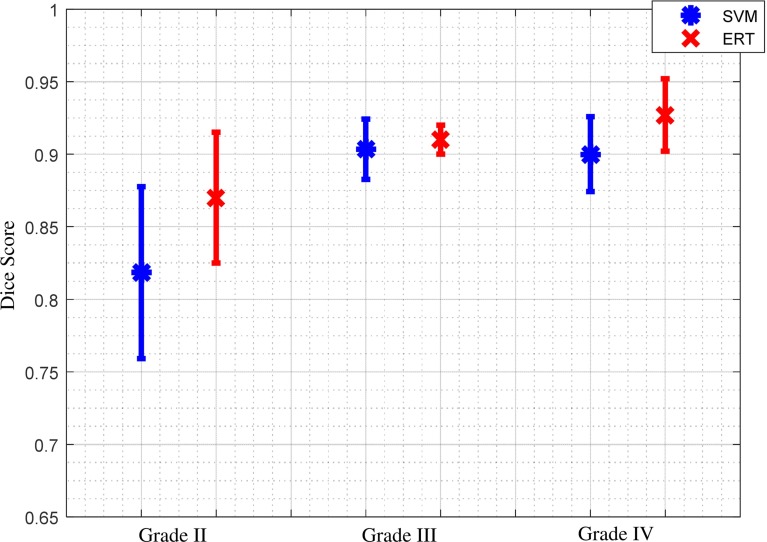
Comparison between average and standard deviation of Dice score overlap measure for SVM versus ERT for different tumour grade types II to IV

Figure 11 shows comparison results of Dice score overlap measure (mean and standard deviation) for SVM versus ERT for different tumour grade types from II to IV. The results show that using ERT classifier increases the segmentation accuracy for all grades of tumour type. There is an evident difference between segmentation overlap measures for different tumour grades using SVM classifier. The result is not satisfactory for grade II (with mean overlap of 0.81), compared with the other two grades (with mean overlap of 0.90), while the segmentation results based on ERT classifiers are consistent for all tumour grade types, with mean overlap of 0.91.

The Wilcoxon signed-rank test is employed to determine whether there are any differences in both the segmentation measure of Dice overlap and classification measures of precision and sensitivity, obtained using the two different classifiers (i.e. SVM and ERT), at 99 % confidence level, with 19 subjects. Our analysis, based on the *p* and *z* values of the statistical test, suggests that there is a statistically significant improvement in the segmentation measures of Dice overlap and in the classification measures of precision and sensitivity, when using the ERT classifier instead of the SVM. Table 5 shows the statistical parameters of our analysis.

**Table 5:** Statistical parameters of the Wilcoxon signed-rank test

	*p*	*Z* value
Dice	<0.001	−3.826
Precision	<0.001	−3.823
Sensitivity	0.001	−3.340

**Fig. 12:**
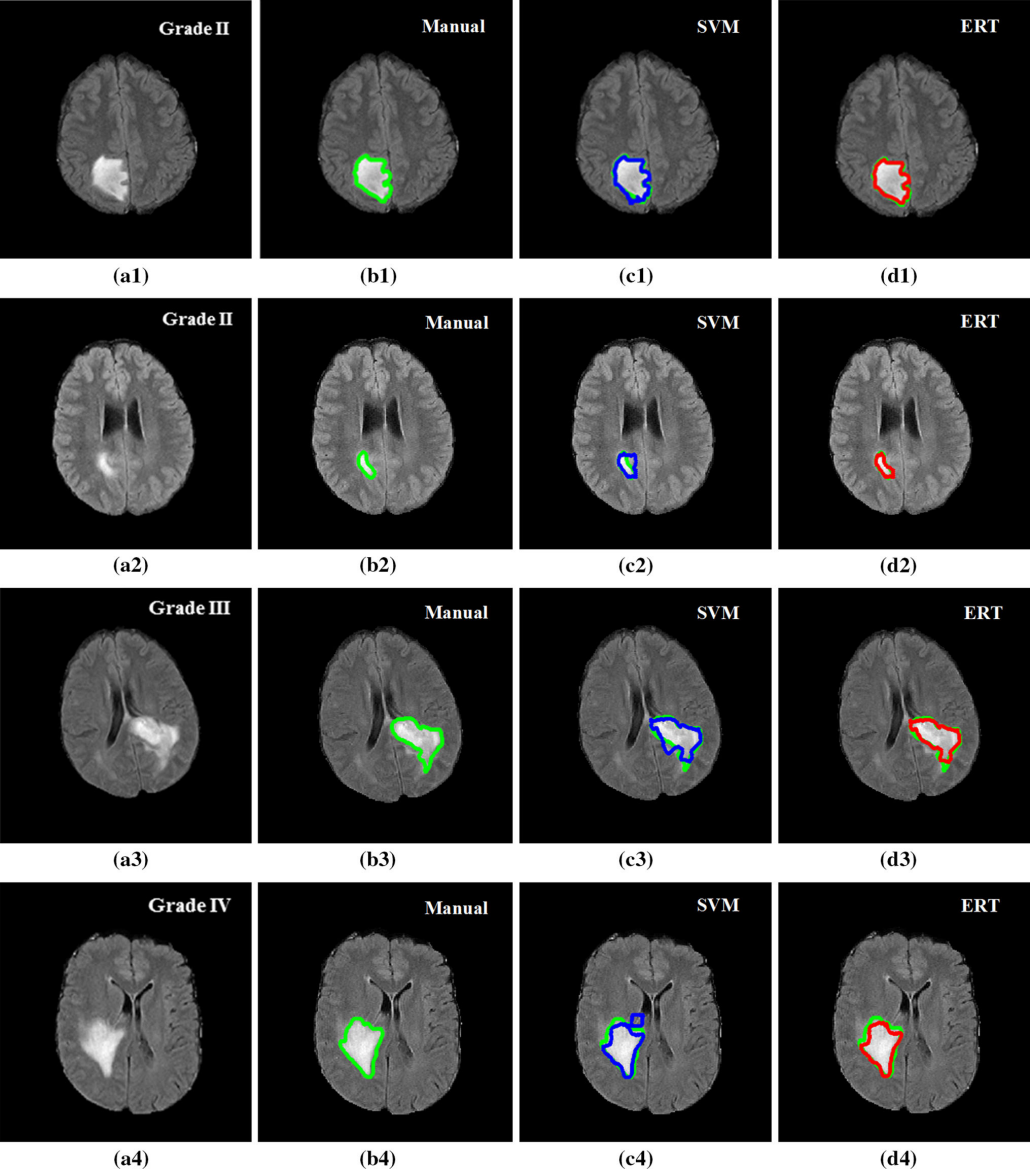
Examples of segmentation results overlay on manual segmentation (*green*). FLAIR image with tumour grade II (*a1*), grade II (**a2**), grade III (**a3**) and grade IV (**a4**); **b1**–**b4** manual segmentation; **c1**–**c4** results using SVM; and **d1**–**d4** results using ERT. Both SVM- and ERT-based methods obtained satisfactory results for the segmentation of different tumour types, with ERT-based method providing slightly better results than that from SVM

**Fig. 13:**
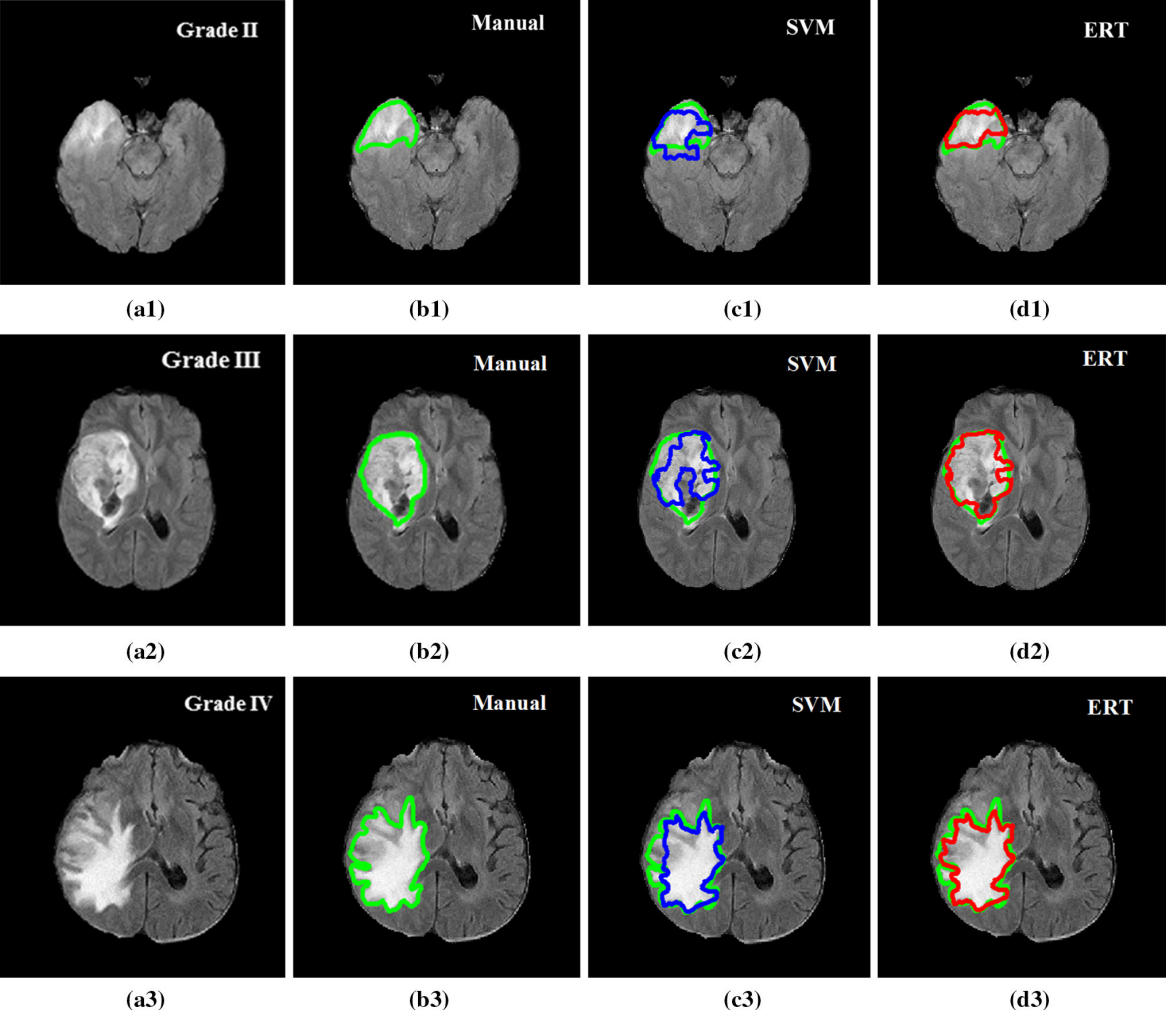
Examples of good detection and segmentation results obtained from ERT-based methods. FLAIR image with tumour grade II (**a1**), grade III (**a2**), grade IV (**a3**); **b1**–**b3** manual segmentation; **c1**–**c3** results using SVM; and **d1**–**d3** results using ERT. Most of the false-positive superpixels from SVM (e.g. (**c1**) and (**c3**)) can be effectively eliminated using ERT, while some tumour superpixels which are wrongly classified to the normal brain tissues by using SVM (e.g. (**c2**)) can be correctly classified as tumour by using the ERT

Figure 12 shows examples of segmentation results for ERT and SVM methods overlaid on the manual annotation. Both SVM- and ERT-based methods obtained satisfactory results for the detection and segmentation of different tumour types, with ERT-based method providing slightly better results than that from SVM. Figure 13 shows examples of much better detection and segmentation results obtained from ERT-based methods, compared to that from SVM. Most of the false- positive superpixels from SVM (e.g. Figs. 12c4 and 13c1) can be effectively eliminated using ERT, while some tumour superpixels which are wrongly classified to the normal brain tissues by using SVM (e.g. Fig. 13c2, c3) can be correctly classified as tumour by using the ERT, demonstrating the higher sensitivity of the ERT. Comparison examples of segmentation for grade II tumour in the first row of both Figs. 12 and 13 illustrate that the segmented tumour boundary from ERT (d1) is closer to the manual annotation, compared to that of SVM (c1).

### Evaluation on BRATS 2012 dataset

To assess the robustness of our method, we further validate the method on a publicly available BRATS 2012 clinical training dataset [8, 9]. In this section, the data are described and the segmentation results are presented and discussed.

#### BRATS 2012 dataset description

The BRATS 2012 annotated clinical training dataset is used which consists of multicontrast MR scans of 30 glioma patients (e.g. 10 low grade and 20 high grade) [7–9]. It should be noted that the BRATS 2012 clinical training datasets are similar to that of BRATS 2013. For those training set, the ground truths are provided by a trained human expert [7]. For each patient data, T1, T2, FLAIR and post-gadolinium T1 MR images are available. Data were acquired from multicentres and using different scanners with different field strengths (1.5 T and 3T). In this study, only FLAIR images are used to evaluate our method.

#### Experimental results

The majority of parameters tuned for our own clinical dataset, including compactness coefficient for superpixel segmentation, fractal features, and number of clusters for texton generation, are directly used in the BRATS dataset. All the parameters for both ERT and SVM classifiers are the same. However, only superpixel size and filter size used for Gabor filter defined in Eq. (4) are slightly adjusted, e.g. for superpixel size, instead of using size of 6 in our own dataset, size of 5 is used in the BRATS dataset, while a smaller range of filter size (e.g. [0.3 0.5 0.8 1.1 1.4]) is used for the Gabor filter bank in texton feature extraction. This is due to the different image sizes and resolutions between the two datasets. All the five features selected using mRMR are also used in BRATS dataset for the classification of each superpixel.

Table 6 presents the evaluation measures for SVM and ERT, respectively. It can be seen that ERT produces a slightly better classification performance, compared to that of SVM, with an overall classification precision of 89.09 %, sensitivity of 88.09 % and BER of 6 % for ERT and of 83.79, 82.72 and 9 % for SVM, respectively.

**Table 6:** Comparison evaluation on superpixel classification using SVM-based and ERT-based classifiers, respectively, on BRATS 2012 dataset using 5 features selected by mRMR

Case no.	Grade/ID	SVM			ERT		
		Precision (%)	Sensitivity (%)	BER	Precision (%)	Sensitivity (%)	BER
1	LG-01	87.68	89.43	0.06	91.84	88.18	0.06
2	LG-02	96.98	88.60	0.06	99.02	92.63	0.04
3	LG-04	75.59	81.95	0.10	78.40	90.67	0.05
4	LG-06	84.57	87.42	0.07	92.15	90.05	0.05
5	LG-08	90.95	83.54	0.09	93.11	91.05	0.05
6	LG-11	89.91	82.67	0.09	91.41	86.78	0.07
7	LG-12	91.42	83.19	0.09	92.18	84.19	0.08
8	LG-13	74.48	79.19	0.11	79.28	85.86	0.08
9	LG-14	83.17	80.37	0.10	88.03	82.58	0.09
10	LG-15	76.15	80.60	0.10	82.64	89.29	0.06
11	HG-01	92.77	92.55	0.04	98.47	95.91	0.03
12	HG-02	83.51	82.15	0.09	90.45	88.62	0.06
13	HG-03	85.46	79.59	0.11	91.31	88.68	0.06
14	HG-04	94.08	89.30	0.06	98.69	90.96	0.05
15	HG-05	78.96	72.06	0.14	83.16	77.70	0.12
16	HG-06	81.54	74.77	0.13	93.13	90.32	0.05
17	HG-07	75.48	79.60	0.11	83.16	87.81	0.07
18	HG-08	87.87	90.58	0.05	89.21	93.88	0.04
19	HG-09	84.78	87.04	0.07	87.56	90.35	0.05
20	HG-10	67.77	65.63	0.18	73.17	71.84	0.15
21	HG-11	90.53	85.68	0.08	92.39	90.21	0.05
22	HG-12	88.58	86.82	0.07	92.08	89.36	0.06
23	HG-13	80.10	84.35	0.08	88.64	89.23	0.06
24	HG-14	84.74	87.99	0.07	88.80	91.76	0.05
25	HG-22	78.21	80.75	0.10	88.79	92.83	0.04
26	HG-24	82.50	85.14	0.08	88.87	87.98	0.07
27	HG-25	82.23	86.08	0.07	90.95	88.16	0.06
28	HG-26	84.41	82.60	0.09	91.71	89.84	0.06
29	HG-27	77.16	72.67	0.14	80.93	75.54	0.13
30	HG-22	82.10	79.19	0.11	93.09	90.42	0.05
Mean	All	83.79	82.72	0.09	89.09	88.09	0.06
STD	All	6.63	5.95	0.03	6.00	5.22	0.03

*BER* balanced error rate

The classification is performed for tumours including oedema and active tumour core versus normal brain tissue

The Dice overlap ratio between the ground truth from manual annotation and the segmented tumour using ERT and SVM classifiers for the BRATS dataset is presented in Table 7. It can be seen that the overlap ratio using the ERT-based method is much better than that of SVM-based for all the three tumour grades, with mean Dice score of 0.88 for ERT-based and 0.83 for SVM-based.

**Fig. 14:**
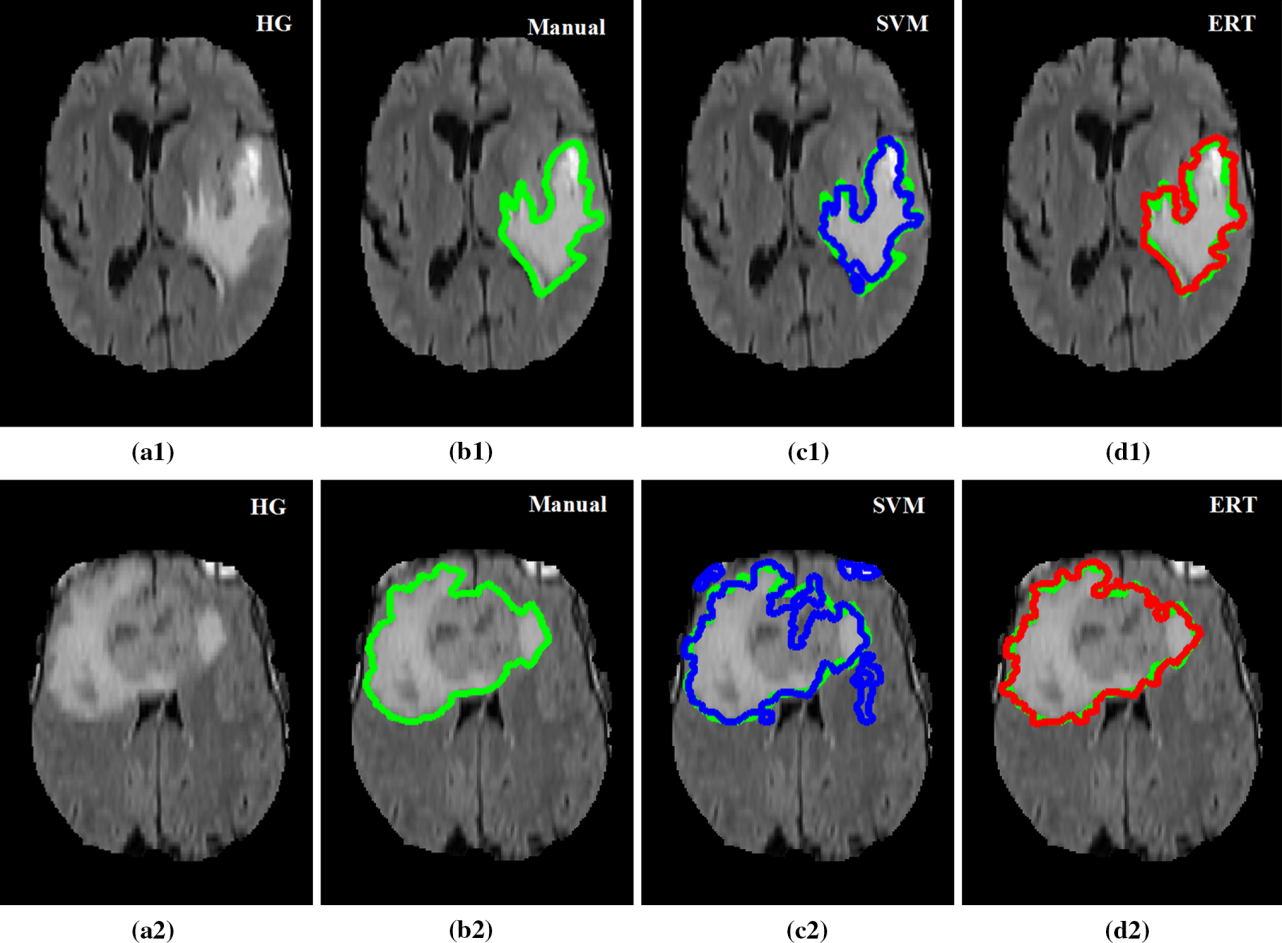
Examples of detection and segmentation results obtained from ERT-based methods on BRATS 2012 data. FLAIR image with high-grade tumour Case HG-01 (**a1**), HG-15 (**a2**); **b1**–**b2** manual segmentation; **c1**–**c2** results using SVM; and **d1**–**d2** results using ERT

**Fig. 15:**
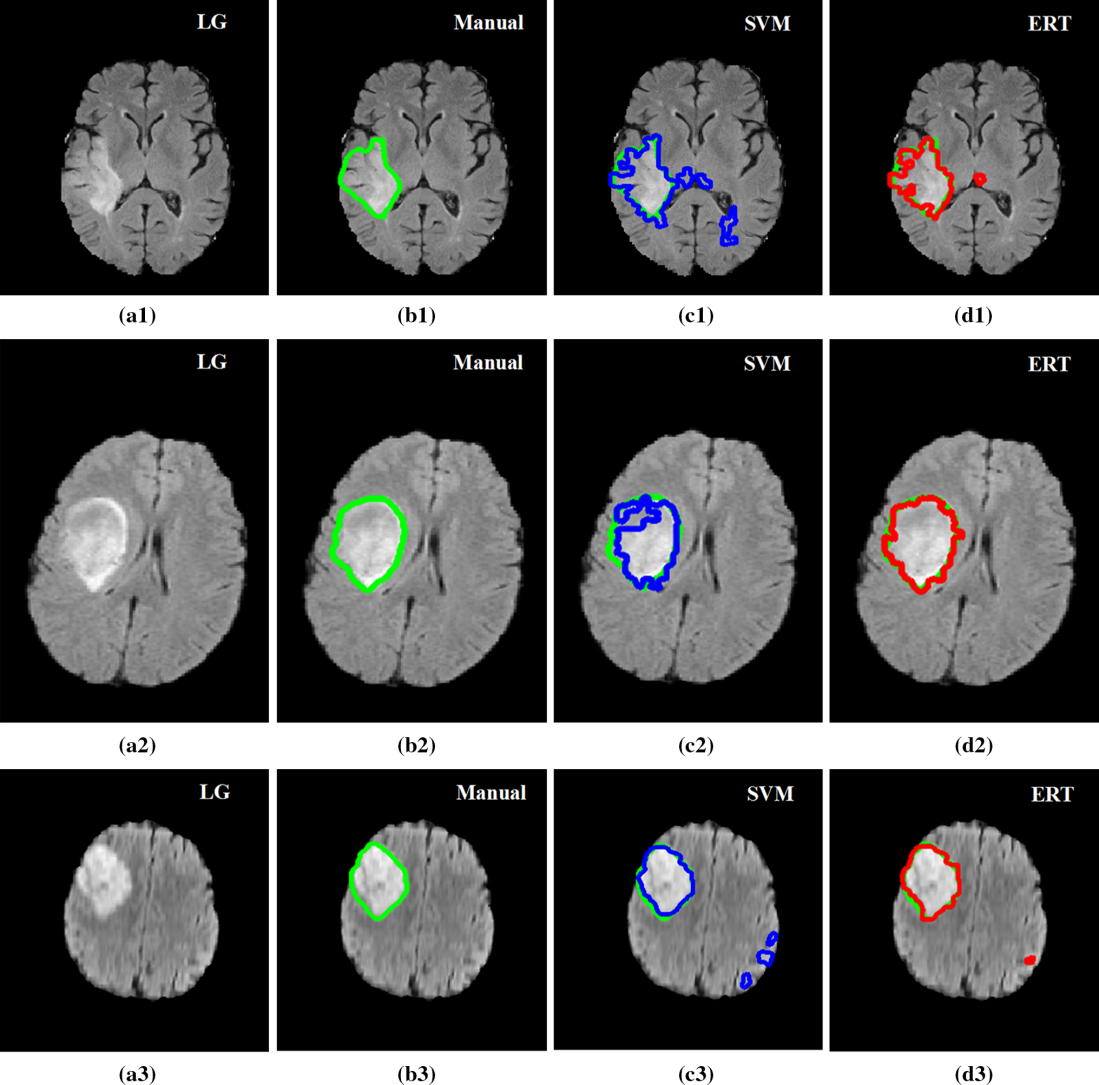
Examples of detection and segmentation results obtained from ERT-based methods on BRATS 2012 data. FLAIR image with low-grade tumour Case LG-04 (**a1**), LG-11 (**a2**) and LG-12 (**a3**); **b1**–**b3** manual segmentation; **c1**–**c3** results using SVM; and **d1**–**d3** results using ERT

Figures 14 and 15 show examples of segmentation results for ERT and SVM methods overlaid on the manual annotations for high-grade tumour (Fig. 14) and low-grade tumour (Fig. 15). Both SVM- and ERT-based methods obtained satisfactory results for the detection and segmentation of different tumour types, with ERT-based method providing slightly better results than that from SVM. Most of the false-positive superpixels from SVM (e.g. Figs. 14c2 and 15c3) can be effectively eliminated using ERT, while some tumour superpixels which are wrongly classified to the normal brain tissues by using SVM (e.g. Fig. 15c2) can be correctly classified as tumour by using the ERT, demonstrating the higher sensitivity of the ERT. Comparison examples of segmentation for both high-grade and low-grade tumours in Figs. 14 and 15 illustrate that the segmented tumour boundary from ERT is closer to the manual annotation compared to that of SVM.

**Table 7:** Comparison results for Dice overlap ratio between manual annotation and the automated segmentation using SVM and ERT for BRATS 2012 dataset (30 scans)

Case no.	Grade/ID	Dice	
		SVM	ERT
1	LG-01	0.85	0.89
2	LG-02	0.93	0.95
3	LG-04	0.78	0.87
4	LG-06	0.84	0.91
5	LG-08	0.88	0.92
6	LG-11	0.86	0.89
7	LG-12	0.88	0.92
8	LG-13	0.75	0.81
9	LG-14	0.80	0.84
10	LG-15	0.78	0.88
11	HG-01	0.89	0.92
12	HG-02	0.83	0.88
13	HG-03	0.82	0.91
14	HG-04	0.90	0.92
15	HG-05	0.74	0.78
16	HG-06	0.79	0.91
17	HG-07	0.78	0.85
18	HG-08	0.89	0.91
19	HG-09	0.86	0.89
20	HG-10	0.65	0.71
21	HG-11	0.87	0.92
22	HG-12	0.88	0.91
23	HG-13	0.81	0.89
24	HG-14	0.86	0.90
25	HG-15	0.78	0.91
26	HG-22	0.84	0.88
27	HG-24	0.85	0.89
28	HG-25	0.84	0.90
29	HG-26	0.75	0.79
30	HG-27	0.81	0.91
Mean	All	0.83	0.88
STD	All	0.06	0.05

## Discussion

### Discussion of applying our method to BRATS dataset

**Fig. 16:**
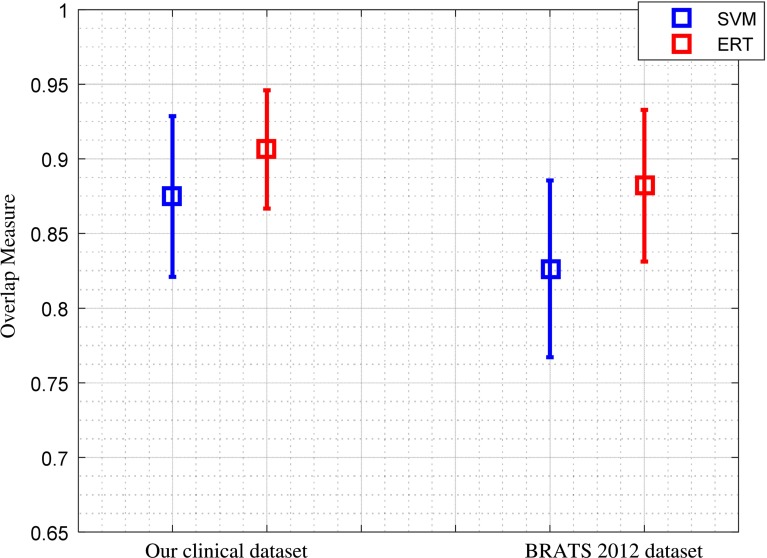
Comparison of the average and standard deviation of Dice score overlap measures for SVM versus ERT for all 19 data scans in our dataset and 30 clinical scans in BRATS 2012 dataset

The BRATS clinical training dataset is used to further evaluate the robustness of the method. As discussed in “Experimental results” section, the majority of the parameters are the same as those optimized for our own clinical data. The overall average and standard deviation of Dice score overlap measures for all our 19 patient data and 30 BRATS 2012 dataset using both ERT-based and SVM-based methods are shown in Fig. 16. The results show that using the state-of-the art ERT for classification of superpixels results in more accurate and robust segmentation compared to that of SVM classifier. For our own clinical dataset, the Dice score overlap measure for ERT-based segmentation is 0.91 ± 0.04, while for SVM-based method, it is 0.87 ± 0.05. For BRATS 2012 dataset, the score overlap measure for ERT-based segmentation is 0.88 ± 0.05, while for SVM-based method, it is 0.83 ± 0.06. It can be seen that the mean Dice scores obtained from BRATS training dataset are closer to that from our own clinical dataset; this suggests robustness of the method.

A comparison of our proposed method on BRATS 2012 clinical dataset with the best scores in the challenges [7] is presented in Table 8. As shown in Table 8, method in Tustison et al. [27] which was the winner of on-site BRATS 2013 challenge was performed on the challenge data. Though datasets might be different, the best on-site score could provide a comparable reference using BRATS dataset. Also, comparing our method to the method by Reza and Iftekharuddin [29] which has the best result for the training set of the BRATS multiprotocol dataset (this is the same dataset used in our evaluation; however, we only use FLAIR protocol), our method has achieved the average Dice overlap of 0.88 which is closer to that of 0.92 by Reza’s method. As discussed in “Experimental results” section, to assess the robustness of our method, the similar optimum parameters and the same five features tuned for our own clinical dataset are directly applied to the BRATS dataset. In particular, our algorithm is trained on 1.5T data from a signal centre, whereas the BRATS data contain multicentre data from 1.5T and 3T MRI scanners and will likely contain variability of image features and contrast that would not be accounted for within our current optimization and training phase.

## Discussion of our method

FLAIR images are routinely acquired in clinical practice as part of standard diagnostic clinical MRI of brain tumours. Our experimental results shown in Tables 4, 5 and Fig. 10 demonstrate high performance of automated detection and segmentation of the brain tumour oedema and core regions in FLAIR MRI. The method was also further validated on BRATS 2012 training dataset (FLAIR) with the similar model parameters and features tuned for our own clinical dataset; good results shown in Tables 6 and 7 suggest the robustness of our method.

**Table 8:** Comparison with other related methods using BRATS dataset (MICCAI 2012)

Method	Description	Comment	Whole tumour (dice)
Tustison et al. [27]	Random forests (ANTs/ANTsR package)	Best MICCAI 2013 on-site	0.87
Reza and Iftekharuddin [29]	Random forests + texture features	Best on training MICCAI 2013	0.92
Our method	ERT + supervoxels	Training MICCAI 2012	0.88

Our method and Reza and Iftekharuddin [29] are performed on BRATS clinical training data and the other work (Tustison et al. [27]) is performed on BRATS challenge data

Selecting an appropriate superpixel size is critical for increasing the overall segmentation accuracy within an optimum calculation speed. Large superpixel size can ensure fast computation and may provide sufficient information for feature extraction such as stable texture features. However, large size of superpixel may contain more than one class of pixels which leads to inaccurate feature calculation (such as small areas of calcification or haemorrhage), and it is also not suitable for small-sized lesions. While small size of superpixel has higher probability of purely containing one class of pixels, it is preferred for small lesion segmentation. However, they may not have enough pixels for calculating stable features, and the computation time for generating the small size partitions is very high. In this study, the size of superpixel is obtained through exhausted parametric searching during the training stage. An optimization algorithm such as genetic algorithm can be explored to effectively find an optimum superpixel size which provides a good trade-off between computation time and segmentation accuracy.

Another important parameter in superpixel segmentation stage is the compactness factor. Higher value of this parameter leads to more rigid partitions which are more stable and usually less noisy, i.e. holes or sparse separated pixels. However, the segmentation may not follow the tissue boundaries very well, especially in the cases where there are no sharp or clear boundaries. While, lower compactness values result in more flexible and accurate boundaries, but the segmentation may produce more isolated and disconnected pixels. They also may generate very narrow superpixels which are not appropriate for texture analysis. In our current study, the compactness factor is determined through visual inspection. Optimization methods need to be investigated to obtain an optimum compactness factor which provides a good trade-off between noise and flexibility.

For the comparison of our method on BRATS data, we refer to the work published in [7] which used these data in MICCAI challenge. However, some of their methods are assessed on the training dataset, while others are on the separate testing dataset. Due to the fact that our current study is based on binary classification (i.e. tumour including oedema and active tumour core versus normal brain tissue) using single FLAIR protocol, it is difficult to have a direct comparison with the current published methods on BRATS data. However, our results which are in the same range of other methods and are close to the best segmentation of whole tumour demonstrate the promise of the method.

Although currently we have only evaluated our segmentation algorithm for FLAIR images, it should be straightforward to apply the same superpixel methodology to contrast-enhanced T1w images and determine the signal intensity and higher-order features that best segment the contrast-enhancing region of high-grade gliomas. In fact, we are currently working on this direction.

In this study, we note the importance of the preprocessing step, namely MRI histogram normalization. This is of particular importance when the method is applied to BRATS dataset, whose data are from multicentres and different scanners.

In the current study, we also note in Fig. 13a2 that small hypointense spots in the FLAIR (and corresponding T1w) may be calcifications, and the hypointense FLAIR region, which is excluded by the SVM method (Fig. 13c2) but included in the ERT analysis (Fig. 13d2), is haemorrhagic since there is hyperintensity in the T1w MRI. This is a limitation of the current single modality analysis if these regions need to be separately specified. Future studies extending our method to multimodal data are planned. This will include the segmentation of different tissue subtypes (e.g. necrosis, active tumour, infiltrative tumour, oedema) by incorporating information from multimodal clinical MRI, including perfusion and diffusion imaging.

For our own clinical dataset, the ground truths were provided based on one expert’s manual annotation. There may have some errors in the manual annotations, which may include intratumoural bleeding or calcification in the tumour (e.g. in Fig. 13b2). When those annotations are used to train the model, it may lead to some errors in the final segmentation. Also, our current clinical dataset mainly contains general cases, such as different tumour grades from a wide range of patient ages (patient ages at the time of scanning ranged from 22 to 73). In the future, we will look into more complicated cases, such as calcification, intratumoural bleeding or elderly patients with white matter disease, which are clinically very important to distinguish against.

## Conclusion

This paper proposed a fully automated method for the detection and segmentation of brain tumour from FLAIR MRI images, by calculating Gabor texton feature, fractal analysis, curvature and statistical intensity features from superpixels. ERT is then used to classify each superpixel into tumour or healthy brain tissue. The formation of superpixel by grouping voxels with similar properties and extracting features from superpixels can not only improve the accuracy of feature extraction, especially for the superpixels near the boundaries between different tissues, but also significantly reduce the computation time, compared to voxel-based feature calculation and classification. The experimental results demonstrate the high detection and segmentation performance of the proposed method using ERT classifier, with average sensitivity of 89.48 %, BER of 6 % and Dice overlap ratio of 0.91. To assess the robustness of the method, the method was further evaluated on BRATS 2012 dataset, which results in similar good performances of 88.09 %, 6 % and 0.88, respectively. This provides a close match to expert delineation across all grades of glioma, leading to a faster and more reproducible method of brain tumour delineation to aid patient management.
